# Secondary research use of personal medical data: attitudes from patient and population surveys in The Netherlands and Germany

**DOI:** 10.1038/s41431-020-00735-3

**Published:** 2020-10-01

**Authors:** Gesine Richter, Christoph Borzikowsky, Wiebke Lesch, Sebastian C. Semler, Eline M. Bunnik, Alena Buyx, Michael Krawczak

**Affiliations:** 1 Institute of Experimental Medicine, Division of Biomedical Ethics, Kiel University, University Hospital Schleswig-Holstein, Kiel, Germany; 2 Institute of Medical Informatics und Statistics, Kiel University, University Hospital Schleswig-Holstein, Kiel, Germany; 3 Technologies, Methods and Infrastructure for Networked Medical Research (TMF e.V.), Berlin, Germany; 4 Department of Medical Ethics, Philosophy and History of Medicine, Erasmus MC, Rotterdam, The Netherlands; 5 Institute of History and Ethics in Medicine, Technical University of Munich, Munich, Germany

**Keywords:** Medical research, Social sciences

## Abstract

Making routine clinical-care-data available for medical research requires adequate consent to legitimize use and exchange. While, public interest in supporting medical research is increasing, individuals often find it difficult to actively enable researchers to access their data. In addition to broad consent, the idea of (consent-free) data donation has been brought into play as another way to legitimize secondary research use of medial data. However, flanking the implementation of broad consent policies or data donation, the attitude of patients, and the general public toward different aspects of these approaches needs to be assessed. We conducted two empirical studies to this end among Dutch patients (*n* = 7430) and representative German citizens (*n* = 1006). Wide acceptance of broad consent was observed among Dutch patients (92.3%), corroborating previous findings among German patients (93.0%). Moreover, 28.8% of the Dutch patients generally approved secondary data-use for non-academic research, 42.3% would make their decision dependent upon the type of institution in question. In the German survey addressing the general population, 78.8% approved data donation without explicit consent as an alternative model of legitimization, the majority of those who approved (96.7%) would allow donated data to be used by universities and public research institutions. This willingness to support contrasted sharply with the fact that only 16.6% would allow access to the data by industry. Our findings thus not only add empirical evidence to the debate about broad consent and data donation, but also suggest that widespread public discussion and education about the role of industry in medical research is necessary in that context.

## Introduction

Increasingly, data from routine clinical care are made available systematically and comprehensively for medical research. Various funding schemes have been implemented to this end in many countries, including the Medical Informatics Initiative (MII) instigated by the German Federal Ministry of Education and Research [1, 2] and recent research data programs funded by the Dutch Association of University Hospitals [3] and the Dutch Ministry of Health, Welfare and Sports [4, 5]. In addition, biospecimens collected in the course of clinical care in The Netherlands are made available to researchers through a nationwide histo- and cytopathology registry (PALGA) [6]. In Germany, biobanks at university hospitals have formed the German Biobank Association (GBA), coordinated by the German Biobank Node (GBN) of the European BBMRI-ERIC initiative, to coordinate the sampling, storage, and provision of research-relevant biospecimens from prospective studies as well as routine clinical care [7]. All these activities immediately raise the issue of adequate patient consent to legitimize the exchange and use of data and biomaterial.

For prospective medical research involving human subjects to be ethically sound, participants need to give prior informed consent (e.g., [8, 9]). This means that the individuals concerned must be informed beforehand about the purpose, nature, risks, and benefits of the study in question, and that they are able to decide about participation voluntarily and without coercion. However, this approach is difficult to translate from prospective clinical studies to the secondary research use of medical data collected in the course of clinical care, where not all potential purposes of the future use of the data can be anticipated at the time of collection. Consequently, there has been a discussion going on for sometime (e.g., [10]) about alternative types of consent suitable for secondary data use that would balance the autonomy of patients and the freedom of research [11]. From an ethical point of view, this dilemma undoubtedly echoes the commonly perceived conflict between individual autonomy and the common good [12].

The concept of so-called “broad consent” is becoming increasingly important for patient-based medical research. In fact, international bodies governing health research, including the World Medical Association and the Council for International Organizations of Medical Sciences/ World Health Organization [8, 9], have approved the implementation of broad consent as “an acceptable alternative” [9, p. 42]. Moreover, in the context of biobanking, research institutions in many countries have already adopted broad consent policies [13]. Following this approach for data as well would not only legitimize a wide, yet underminable scope of research use in the future but could also facilitate comprehensive data sharing within the research community, if the consent process was designed accordingly. This way, broad consent would address the many relevant demands with regard to openness and transparency, for example in the form of the FAIR principles [14] or through corresponding statements by the International Committee of Medical Journal Editors (ICMJE) [15].

At the same time, public interest in supporting medical research through the provision of access to medical data increases [16–18]. Vis-a-vis the growing amount and scientific potential of such data, however, this development led to an ethically problematic asymmetry: On the one hand, the importance of data for improving population health is widely acknowledged. At the same time, individuals find it difficult to enable researchers directly to actually access their data, which infringes upon their decision autonomy if they want their data to be used [19]. In view of this misalignment, the German Ethics Council (Deutscher Ethikrat) recently brought into play the concept of “data donation” as a way forward for individuals to facilitate research use of their medical data [20, p. 266f]. Like others, the German Ethics Council understands “data donation” as giving consent to the use of one’s data without limiting its timing and purpose provided that (a) the possible consequences, especially for family members, are made sufficiently clear and (b) an appropriate infrastructure for managing and protecting the data is in place.

The discussion about adequate forms of consent to research data use gained momentum when the EU General Data Protection Regulation (Regulation 2016/679 EU, EU-GDPR) became enforceable law in May 2018. According to Article 6 of the EU-GDPR, processing of personal medical data is forbidden unless (a) the data subject (i.e., the patient) has given consent or (b) the processing (i.e., the research) is carried out in the public interest and under conditions laid down by EU or member state law [21]. However, Section 2(j) of Article 9 of the EU-GDPR allows processing of personal data also if “necessary for archiving purposes in the public interest, scientific or historical research purposes or statistical purposes” [21]. This exemption from prior consent is given in accordance with Article 89, which explicitly leaves room for national law to provide derogations under the requirement of safeguards [ibid.].

Against the above background, it appears worthwhile exploring whether data donation (as defined, for example, by the German Ethics Council) represents an acceptable and adequate alternative form of consent, arguing that with its intention to actively support medical research, data donation clearly has the common good as its main foundational ethical principle [12, 22, 23]. With the results of the two studies reported upon in the following, we hope to be able to contribute further to the exploration of new (and possibly more adequate) forms of consent in medical research.

## Methods

### Background

The present work comprises two surveys, carried out in Germany and The Netherlands in 2019, that addressed the attitude of different societal groups toward the secondary research use of personal medical data under certain consent frameworks.

Both surveys drew upon two earlier studies of ours from Northern Germany. The first one, carried out in 2015, was questionnaire-based and comprised 700 outpatients of the Comprehensive Center for Inflammation Medicine (CCIM), University Hospital Schleswig-Holstein (UKSH) Campus Kiel. The study was mostly concerned with the understanding and acceptance of, and motivation to give, broad consent for healthcare-embedded biobanking [17]. The second study, from 2018, was conducted under the same conditions and assessed the level of acceptance of a no-consent policy to legitimize secondary data use in medical research. Both studies revealed that German patients were not only willing to give broad consent but were also likely, under certain circumstances, to accept use of their medical data for research without explicit consent [18].

As a first step toward generalizing our earlier results to other countries and other societal groups, we assessed the attitude toward, and understanding of, broad consent to a large sample of Dutch individuals from outside the clinical context (“Acceptance of broad consent in The Netherlands” section). Notably, Dutch university hospitals have traditionally followed an “opt out” policy to legitimize secondary research use of medical data and biospecimens. However, first hospitals have recently introduced [24], or are currently piloting, broad consent (“opt in”) solutions. In a second, representative population survey, we asked members of the general public in Germany for their attitude toward data donation, i.e., toward the practical implementation of a no-consent policy (“Attitude toward data donation without explicit consent in Germany” section).

### Statistics

Descriptive statistics were calculated using IBM SPSS Statistics for Windows [25]. For categorical variables, e.g., the agreement, or not, to a given statement, we determined absolute as well as relative frequencies and used χ^2^ or Fisher’s exact tests, as appropriate, to assess the statistical significance of any differences noted between Germany and The Netherlands. *P* values smaller than 0.05 were regarded as statistically significant.

## Acceptance of broad consent in The Netherlands

### Materials and methods

In co-operation with Patiëntenfederatie Nederland and with support of the Dutch Ministry of Health, Welfare, and Sports, we conducted a web-based survey among 22,000 Dutch patients in 2019, using a questionnaire comparable to that employed in two previous German surveys [17, 18]. In addition, patients were provided an information brochure and a (hypothetical) consent form that represented near-verbatim translations of the corresponding documents routinely issued at UKSH Campus Kiel, Germany, with minor modifications of language, including more informal and simpler language as well as shorter sentences. The only notable difference with regard to content concerned the policy of reporting incidental findings, which had to be re-phrased for the Dutch study in order to comply with national law.

In contrast to the two German surveys, where the attitude toward broad consent was evaluated in the clinic, the goal of the Dutch study was to find out whether patients would accept broad consent even if only affecting them sometime in the future. After answering some general questions about their views on using personal data and biospecimen for scientific research, patients had the choice to read the informational brochure and consent form as well. Patients who did so were then asked to answer questions assessing their understanding of the documents and their attitude toward the approach, and whether this attitude had changed after reading the documents.

As in the German surveys, Dutch patients were also asked to rate their own social attitude. To this end, the German version of the Short Schwartz’s Value Survey (SSVS) [26] was translated into Dutch. The SSVS is a well-established tool world-wide [27, 28] and has been validated before in its German version [29]. The underlying theory assumes the existence of ten basic human values (power, achievement, hedonism, stimulation, self-direction, universalism, benevolence, tradition, conformity, and security) that can be identified across most human societies and that shape behavior and decision-making of human individuals.

### Results

#### Acceptance of broad consent

Of the 22,000 Dutch patients approached, 7430 participated in the web-based survey (33.8%) and 5258 (70.8% of those who participated) also read the (hypothetical) broad consent documents, followed by completion of the corresponding part of the survey. The study thus achieved an acceptable level of participation for its type (i.e., questionnaire delivery). Most patients were over 60 years of age and highly educated [details were published before by Patiëntenfederatie Nederland, 30] but, since age and education were not found to be of any significant influence on the attitude of patients in the previous German surveys [17, 18], the Dutch and German studies should be well comparable. Of the 5258 participants who agreed to regard the broad consent documents, a total of 3708 (70.5%) had also revealed their attitude toward research use of their data earlier in the survey. Some 3422 of these (92.3%) expressed subsequent willingness to give broad consent, suggesting an approval rate among Dutch patients that is comparable to that noted in the German surveys [17, 18].

#### Understanding of broad consent

Objective understanding of the broad consent approach was better in the Dutch than in the 2016 German survey (Table 1) despite the fact that the German documents were developed further during the survey. Significantly higher proportions of correct answers (*p* < 0.05) were given to questions concerning the scientific scope of data and biospecimen use (54.1%), the right to withdraw (76.8%), the possible use of data and biospecimen by external researchers (59.0%), the lack of absoluteness of data protection (84.5%), and the lack of immediate personal benefit from consenting (91.6%). Only with regard to the policy for reporting incidental findings, i.e., the only item where the contents of the Dutch information brochure differed from the German, understanding was significantly poorer among Dutch patients (54.5% vs. 65.0% for Dutch and German patients, respectively; Table 1).

**Table 1:** Understanding of broad consent by Dutch and German patients.

Topic	German survey 2016^d^ (*n* = 254) Number (%)	Dutch survey 2019 (*n* = 5258) Number (%)	*P* value
Reporting of incidental findings^a^	165 (65.0)	2865 (54.5)	0.001
Scientific scope of use^b^	117 (46.1)	2846 (54.1)	0.012
Right to withdraw^b^	164 (64.6)	4036 (76.8)	<0.001
Use by external researchers^b^	81 (31.9)	3103 (59.0)	<0.001
Absoluteness of data protection^c^	142 (55.9)	4443 (84.5)	<0.001
Personal benefit	170 (66.9)	4815 (91.6)	<0.001

^a^The information brochures described different policies because of country-specific legal concerns regarding the reporting, or not, of incidental findings.

^b^This topic was addressed by the provision of two contradictory statements; incorrect affirmation of at least one statement or non-affirmation of both statements was counted as a wrong answer.

^c^The wording of the German and Dutch question differed slightly.

^d^Data refer only to phase 2 of the 2016 German survey because the brochure used there underwent intermittent linguistic revision.

#### Motivation and human values

In the 2018 German survey [18], most participants who gave broad consent did so mainly for pro-social reasons, including altruism, reciprocity, solidarity, and gratitude (Table 2). In the Dutch survey reported here, support of research in general was also found to be the main reason for giving broad consent (*n* = 1375, 40.2%), although at a substantially lower level than in the Germany survey (86.1%). All other aspects of altruism, solidarity, and reciprocity were prevalent among Dutch patients at a consistent albeit low level (20–25%), and gratitude seemed to play an even more minor role (*n* = 487, 14.2%; Table 2). The predominant SSVS human value in both the Dutch and the German 2018 survey was benevolence. While security was by far the second most important value for German participants, security, universalism, and self-direction played an almost equally important role among the Dutch (data not shown).

**Table 2:** Motivation to give broad consent.

Concept	Motivational item	German survey 2018 (*n* = 468) Number (%)	Dutch survey 2019 (*n* = 3422) Number (%)	*P* value
Altruism	Support of research in general	403 (86.1)	1375 (40.2)	<0.001^a^
	Helping all future patients	315 (67.3)	859 (25.1)	<0.001^a^
Reciprocity	Returning own benefit from research	339 (72.4)	794 (23.2)	<0.001^a^
Solidarity	Helping future patients with same disease	328 (70.1)	699 (20.4)	<0.001^a^
	Feeling connected with future patients	189 (40.4)	848 (24.8)	<0.001^a^
Gratitude	Gratitude toward doctors	214 (45.7)	487 (14.2)	<0.001^a^
Other	Hope for personal benefit	153 (32.7)	847 (24.8)	<0.001^a^
	Knowing of others who consented	16 (3.4)	22 (0.6)	<0.001^b^
	Worry about disadvantages when not consenting	4 (0.9)	18 (0.5)	0.328^b^
	No specific reasons	41 (8.8)	21 (0.6)	<0.001^b^

^a^χ^2^-test

^b^Fisher’s exact test.

#### Attitude toward secondary use of medical data for purposes other than academic research

**Fig. 1:**
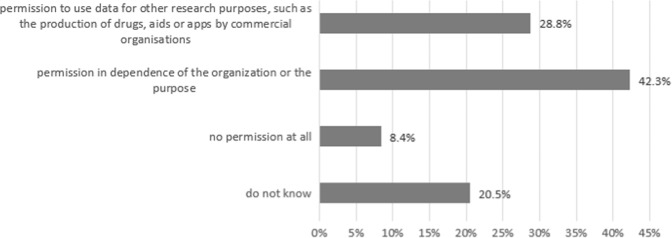
Attitude of Dutch patients toward secondary use of medical data for purposes other than academic research (*n* = 7430). Answering behaviour to the following question: “Data can also be used for other types of research, e.g. to produce medicine, tools or apps by commercial organisations. Would you permit the use of your data for those purposes?”.

Inspired by critical aspects of the German study on data donation reported in Materials and methods in “Attitude towards data donation without explicit consent in Germany” section below, the Dutch questionnaire also asked participants for their attitude toward the secondary use of medical data for purposes other than academic research, including the commercial development of pharmaceuticals or apps. While 2139 patients (28.8%) fully approved and 624 (8.4%) entirely disapproved such use (Fig. 1), the willingness of 3146 patients (42.3%) to grant permission for secondary data use was found to depend upon the nature of the beneficiary institution or the purpose of the data usage. A total of 1521 patients (20.5%) were indifferent toward the issue (“do not know”).

## Attitude toward data donation without explicit consent in Germany

Our previous 2018 German survey [18] was carried out shortly before the EU-GDPR came into force. The goal of that study was to assess the attitude of patients toward the EU-GDPR stipulation that, under certain conditions, patients no longer would have to consent to the use of their personal data for scientific research. The main outcome of the survey, namely that a considerable majority of participants (75.7%) approved of such a consent-free approach, brought into play the possibility of data donation as an alternative form of legitimizing the secondary research use of medical data. In order to assess the generalizability of the survey results to non-patients, a population-based study on the topic was instigated in 2019 by TMF e.V. (Technologies, Methods and Infrastructure for Networked Medical Research), an umbrella non-profit organization for medical research based in Berlin, Germany.

### Materials and methods

The aim of the 2019 TMF survey reported here was to evaluate the attitude of the general population in Germany toward medical data donation for research purposes. It was conceptualized and carried out against the background of almost 20 years of experience, by TMF, of negotiating with data protection authorities and ethics committees in the different German federal states. The goal of these consultations, namely a uniform broad consent for all states, turned out to be a critical prerequisite for the German Medical Informatics Initiative (MII), launched in 2017 by the federal government and coordinated by TMF. The 2019 survey on data donation also marked the beginning of a comprehensive dialogue between the German university hospitals, represented by the MII, and patient organizations as well as self-help groups, which are well organized in Germany and have a strong interest in medical research. The survey was designed as a standardized telephone questionnaire of a representative population sample (*n* = 1006) and was conducted by the German Forsa Institute between 13 and 18 August 2019. The study sample was drawn from Forsa’s population-representative survey panel “forsa.omninet”. The survey included three questions: (i) attitude toward data donation from digital health records, (ii) possible beneficiaries of data donation, and (iii) duration of research use after data donation.

### Results

**Fig. 2:**
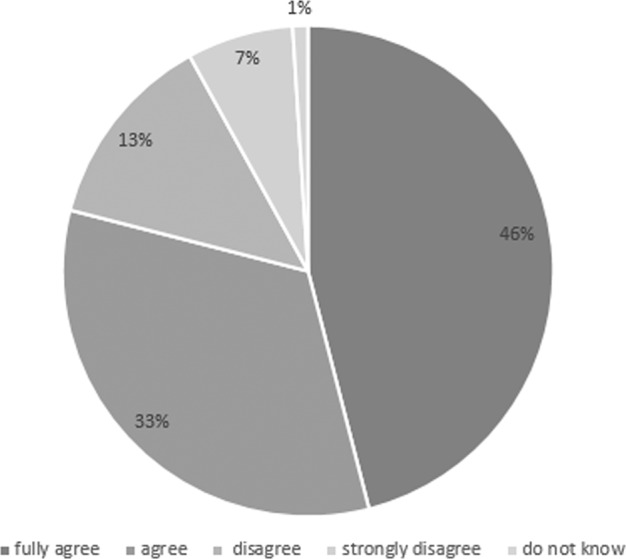
Attitude in the German population toward data donation from digital health records. The pie chart illustrates the answering behavior of the forsa omninet interviewee panel (*n* = 1006) to the following question: “Would you agree your personal health information to be shared anonymously and free of charge for medical research so that diseases can be better diagnosed and new treatments developed in the future?”.

Of the 1006 participants, a majority of 793 (78.8%) approved donation of data from their digital health records as well as sharing of these data with third parties for medical research, anonymously and free of charge. A small minority of participants disagreed or strongly disagreed (*n* = 204, 20.3%; Fig. 2) mainly because of worries about data security (*n* = 122, 58.8%). Only nine participants (0.9%) were indifferent toward the issue (“do not know”).

**Fig. 3:**
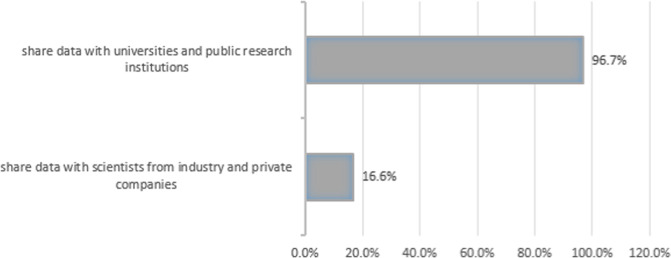
Possible beneficiaries of data donation for medical research. Answering behaviour (based upon forsa omninet respondents to question 1 who fully agreed, or agreed, to data donation; *n* = 793) to the following question: “And who would you share your personal health data for medical research with?” Note that percentages do not add up to 100 because multiple answers were possible.

A vast majority of those who approved data donation would allow their data to be used by scientists from universities and public research institutions (*n* = 767, 96.7%), but only a minority (*n* = 132, 16.6%) of mostly younger respondents would also share their health data with scientists from industry and private institutions, including pharmaceutical and biotech companies (Fig. 3).

When asked for how long their personal health data should be available for medical research after data donation, a majority agreed to long-term data use. Thus, 440 of the 793 approving participants (55.5%) responded that their data could be used for an unlimited period of time whereas 133 (16.8%) wanted to see research limited to the next five years. To be asked for re-consent for each individual research project was preferred by 212 individuals (26.7%).

## Limitations

We observed significantly better understanding of the broad consent issues in the Dutch survey, compared to the 2016 study in Germany [17], likely due to the overall higher educational level in the Dutch sample, which may point toward a lack of comparability as a limitation of our current study. However, public awareness of the issues of secondary data use for medical research has improved in the recent past (e.g., [31]). Therefore, the different levels of comprehension could also be due to the fact that the topic has received more public attention over the last three years in The Netherlands, thereby resulting in better prior knowledge among the Dutch patients. In any case, it is not likely that the Dutch had better prior knowledge because, of those Dutch patients who did not change their opinion (*n* = 3562) upon reading the broad consent brochure, only 21.1 % (*n* = 751) claimed to have known the information beforehand. Another possible cause of the different understanding in different countries may be that the documents were translated from German into slightly easier Dutch language. This explanation receives further credit from our 2016 study in Germany [17] where linguistic simplification improved the understanding of the information material as well.

## Discussion

As a means to legitimize the secondary research use of medical data, broad consent policies are becoming adopted more and more widely in Germany and The Netherlands. At the same time, however, the creation of ever more complex infrastructures for data integration from different sources, as currently exemplified by the German Medical Informatics Initiative, calls for further development of the consent process. Although there seems to be wide expert agreement on the preconditions for responsible and, hence, ethically acceptable data sharing, including the protection of privacy, minimization of risks, data security, transparency as well as public information and trust [32], little is known about the attitude of the data subjects themselves [31, 33].

The two surveys reported upon here provide further insight into the attitude of data subjects in Germany and The Netherlands towards comprehensive secondary research use of their data, either under a broad consent opt-in policy or an explicit opt-out policy (“data donation”). In both surveys, we observed a strong positive attitude towards supporting medical research in general, corroborating previous studies [32]. This positive attitude, which mainly reflects the human values of benevolence and security, should form a robust basis for reconciling the rights of data subjects with the needs of data-driven medical research.

The general concordance of their outcome notwithstanding, there are some notable differences between the German and Dutch studies. Whereas the German patients were approached in the waiting rooms of a university hospital, the Dutch study was web-based. In addition to linguistic and educational factors (see above), the significantly better understanding of the broad consent approach by Dutch patients may thus be attributable partly to these different interview settings.

When patients are expecting a serious diagnosis or waiting for an unpleasant or even dangerous intervention, they may simply not be receptive to the information provided. This calls for means to inform patients in a situation that is less stressful and burdensome for them, i.e., before they actually become patients. Our findings support the view that informed data donation in a non-clinical context may be an acceptable alternative type of legitimization for the secondary research use of medical data. In fact, the 2018 German patient survey and, even more so, the recent population survey by TMF revealed a generally positive attitude towards data donation for research by way of an opt-out policy, in line with other reports [34].

Both surveys also revealed that, whereas universities and public research institutions seem to enjoy great public trust, data use for commercial research of data is seen more critically by patients and the general population alike. This result corroborates previous work framing the participation in data-rich research as an activity geared towards solidarity and the common good [12]. Moreover, transparency and public involvement are essential for the general acceptance of such research [35], and the failure to implement the “care.data” program in England, aimed at extracting anonymised patient data from GP records in order to build a central nationwide database, is proof to the point in this regard [36]. In addition to the lack of patient awareness of the program, the fact that the database was intended to be accessible by third parties, including pharmaceutical companies, was a major cause of serious public concerns [36]. In the discussions why “care.data” failed, and which future lessons could be learned from this setback, particular attention was paid to the concept of “social license”, whereby public acceptance of health research rests on its status as a socially valuable service to the public good [37]. Such “social licenses” for the research use of patient data cannot be taken for granted but need to be acquired, particularly by the private sector, taking into account the diverse patient and public values, needs and interests [32].

In summary, we observed a generally positive public attitude in Germany and The Netherlands towards data donation for medical research, even without explicit consent. At the same time, however, we recognized considerable reluctance towards research use of the donated data by commercial institutions. Such resentments are not only difficult to take into account in real life, where the collaboration between publicly funded and commercial research is a reality also encouraged by many funding organizations. It also fails to acknowledge the critical importance of economic activity for societal progress. To overcome this incongruity in public attitude, we postulate that more widespread public discussion and education about the role of industry in medical research is necessary, particularly in the context of data donation.

## Supplementary information

Folder

Survey „data donation“ for medical research

Delen van uw data in het ziekenhuis

## References

[1] Medizininformatik initiative https://www.medizininformatik-initiative.de/en/start (2020).

[2] Semler SC, Wissing F, Heyder R (2018). German medical informatics initiative. Methods Inf Med.

[3] Registratie aan de bron. Registratie aan de bron: Zorginformatie delen en optimaliseren. 2020. https://www.registratieaandebron.nl. Accessed 23 Apr 2020.

[4] Ministry of Health, Welfare and Sports. Data voor Gezondheid. 2020. https://www.datavoorgezondheid.nl. Accessed 23 Apr 2020.

[5] Minister of Medical Care dhr. B.J. Bruins. Data laten werken voor gezondheid: Een kwestie van gewaarborgd vertrouwen. Appendix to a Letter to the Chair of the House of Representatives. The Hague, 15 November 2018. https://www.eerstekamer.nl/overig/20181115/data_laten_werken_voor_gezondheid/meta. Accessed 23 Apr 2020.

[6] The nationwide network and registry of histo- and cytopathology in the Netherlands (PALGA Foundation). PALGA. https://www.palgaopenbaredatabank.nl. Accessed 29 Jun 2020.

[7] BBMRI. http://www.bbmri.de. Accessed 29 Jun 2020.

[8] WMA. Declaration of Helsinki – Ethical principles for medical research involving human subjects. 2013. https://www.wma.net/policies-post/wma-declaration-of-helsinki-ethical-principles-for-medical-research-involving-human-subjects. Accessed 22 Apr 2020.

[9] Council for International Organizations of Medical Sciences (CIOMS), in collaboration with World Health Organization. International Ethical Guidelines for Health-Related Research Involving Humans. Geneva, Switzerland: CIOMS, 2016, guidelines 11 and 12. https://cioms.ch/wp-content/uploads/2017/01/WEB-CIOMS-EthicalGuidelines.pdf. Accessed 10 Mar 2020. 40523065

[10] Ploug T, Holm S. The ‘Expiry Problem’ of broad consent for biobank research—and why a meta consent model solves it. J Med Ethics. 2020. 10.1136/medethics-2020-106117 10.1136/medethics-2020-106117 32098907

[11] Richter G, Buyx A (2016). Breite Einwilligung (broad consent) zur Biobank-Forschung – die ethische Debatte. Ethik Med.

[12] Prainsack B, Buyx A (2017). Solidarity in biomedicine and beyond.

[13] Rothstein MA, Knoppers BM (2016). Part II: Harmonizing privacy laws to enable international biobank research. J Law Med Ethics.

[14] Wilkinson MD, Dumontier M, Aalbersberg IJ, Appleton G, Axton M, Baak A (2016). The FAIR guiding principles for scientific data management and stewardship [published correction appears in Sci Data. 2019;:6]. Sci Data.

[15] Taichman DB, Backus J, Baethge C, Bauchner H, de Leeuw PW, Drazen JM (2016). Sharing clinical trial data: a proposal from the International Committee of Medical Journal Editors. PLoS Med.

[16] Nuffield Council on Bioethics. The collection, linking and use of data in biomedical research and health care: ethical issues, London 2015. www.nuffieldbioethics.org/publications/p3. Accessed 05 Mar 2020.

[17] Richter G, Krawczak M, Lieb W, Wolff L, Buyx A (2018). Broad consent for health care-embedded biobanking: understanding and reasons to donate in a large patient sample. Genet Med.

[18] Richter G, Borzikowsky C, Lieb W, Schreiber S, Krawczak M, Buyx A (2019). Patient views on research use of clinical data without consent: legal, but also acceptable?. Eur J Hum Genet.

[19] Krutzinna J, Floridi L. Ethical medical data donation: a pressing issue. In: Krutzinna J, Floridi L (eds) The ethics of medical data donation. Philosophical Studies Series, 2019, vol 137. Springer, Cham, 1:7. 32091864

[20] German Ethics Council. Big Data and Health—Data Sovereignty as Informational Freedom. November 2017.

[21] Regulation (EU) 2016/679 of the European Parliament and of the Council of 27 April 2016 on the protection of natural persons with regard to the processing of personal data and on the free movement of such data, and repealing Directive 95/46/EC (General Data Protection Regulation), http://data.europa.eu/eli/reg/2016/679/2016-05-04.

[22] Krutzinna J, Taddeo M, Floridi L (2019). Enabling posthumous medical data donation: an appeal for the ethical utilisation of personal health data. Sci Eng Ethics.

[23] Prainsack B, Data Donation: how to resist the iLeviathan. In: Krutzinna J, Floridi L, editors. The ethics of medical data donation. Philosophical Studies Series. Cham: Springer, Cham; 2019. vol 137. 12:22. 32091859

[24] Antoni van Leeuwenhoek. Netherlands Cancer Institute (NKI). Toestemming wetenschappelijk onderzoek. 2020. https://www.avl.nl/ons-onderzoek-het-nederlands-kankerinstituut/toestemming-wetenschappelijk-onderzoek. Accessed 23 Apr 2020.

[25] IBM [IBM SPSS Statistics]. Release 22.0.0.2 for Windows, Armonk, NY: IBM.

[26] Boer D. Short Schwartz’s Value Survey in German (SSVS-G). 2013. https://www.uni-koblenzlandau.de/de/koblenz/fb1/institutpsychologie/abteilungen/sozial%20und%20organisationspsychologie/SSVS-G. Accessed 10 Sep 2018.

[27] Yeganeh H, Su Z, Sauers D (2009). The applicability of widely‐employed frameworks in cross‐cultural management research. J Acad Res Econ.

[28] Spini D (2003). Measurement equivalence of 10 value types from the Schwartz value survey across 21 countries. J Cross Psychol.

[29] Boer D. SSVS-G. Short Schwartz’s Value Survey-German. In: Kemper C, Zenger M, Brähler E, editors. Psychologische und sozialwissenschaftliche Kurzskalen. Berlin, Germany: Medizinisch Wissenschaftliche Verlagsgesellschaft; 2014. p. 299–302.

[30] Patientenfederatie Nederland, Delen van uw data, https://www.patientenfederatie.nl/images/stories/dossier/pers_gez_omg/Definitieve_rapportage_delen_van_uw_data.pdf. Accessed 08 Jan 2020.

[31] Howe N, Giles E, Newbury-Birch D, McColl E (2018). Systematic review of participants’ attitudes towards data sharing: a thematic synthesis. J Health Serv Res Policy.

[32] Kalkman S, van Delden J, Banerjee A, Tyl B, Mostert M, van Thiel G. Patients’ and public views and attitudes towards the sharing of health data for research: a narrative review of the empirical evidence. J Med Ethics. 2019. 10.1136/medethics-2019-105651. 10.1136/medethics-2019-105651 PMC8717474 31719155

[33] Xafis VG, Schaefer O, Labude MK, Brassington I, Ballantyne A, Lim HY (2019). An ethics framework for big data in health and research. Asian Bioeth Rev.

[34] Boulos D, Morand E, Foo M, Trivedi JD, Lai R, Huntersmith R (2018). Acceptability of opt-out consent in a hospital patient population. Int Med J.

[35] Langhof H, Kahrass H, Sievers S, Strech D (2017). Access policies in biobank research: what criteria do they include and how publicly available are they? A cross-sectional study. Eur J Hum Genet.

[36] Meszaros J, C Ho C (2019). Building trust and transparency? Challenges of the opt-out system and the secondary use of health data in England. Med Law Int.

[37] Carter P, Laurie GT, Dixon-Woods M (2015). The social licence for research: why care.data ran into trouble. J Med Ethics.

