# Correction: Secondary research use of personal medical data: attitudes from patient and population surveys in The Netherlands and Germany

**DOI:** 10.1038/s41431-021-00971-1

**Published:** 2021-09-30

**Authors:** Gesine Richter, Christoph Borzikowsky, Wiebke Lesch, Sebastian C. Semler, Eline M. Bunnik, Alena Buyx, Michael Krawczak

**Affiliations:** 1 Institute of Experimental Medicine, Division of Biomedical Ethics, Kiel University, University Hospital Schleswig-Holstein, Kiel, Germany; 2 Institute of Medical Informatics und Statistics, Kiel University, University Hospital Schleswig-Holstein, Kiel, Germany; 3 Technologies, Methods and Infrastructure for Networked Medical Research (TMF e.V.), Berlin, Germany; 4 Department of Medical Ethics, Philosophy and History of Medicine, Erasmus MC, Rotterdam, The Netherlands; 5 Institute of History and Ethics in Medicine, Technical University of Munich, Munich, Germany

**Keywords:** Medical research, Social sciences

Correction to: *European Journal of Human Genetics* 10.1038/s41431-020-00735-3

The article “Secondary research use of personal medical data: attitudes from patient and population surveys in The Netherlands and Germany”, written by Gesine Richter, et al., was originally published electronically on the publisher’s internet portal on 1 October 2020 without open access. With the author’ decision to opt for Open Choice the copyright of the article changed on 21 September 2021 to © Author(s) 2020 and the article is forthwith distributed under a Creative Commons Attribution 4.0 International License, which permits use, sharing, adaptation, distribution and reproduction in any medium or format, as long as you give appropriate credit to the original author(s) and the source, provide a link to the Creative Commons licence, and indicate if changes were made. The images or other third party material in this article are included in the article’s Creative Commons licence, unless indicated otherwise in a credit line to the material. If material is not included in the article’s Creative Commons licence and your intended use is not permitted by statutory regulation or exceeds the permitted use, you will need to obtain permission directly from the copyright holder. To view a copy of this licence, visit http://creativecommons.org/licenses/by/4.0.

The original article has been corrected.

