# Supplementary material for: Secondary research use of personal medical data: attitudes from patient and population surveys in The Netherlands and Germany
Source: Eur J Hum Genet. 2020 Oct 1;29(3):495–502. doi: 10.1038/s41431-020-00735-3 (PMC7940390; doi:10.1038/s41431-020-00735-3)
Supplement: Supplementary file 1 — Folder [file 41431_2020_735_MOESM1_ESM.docx]

**Folder**

Voor medisch onderzoek zijn gegevens en lichaamsmateriaal nodig. Bij lichaamsmateriaal gaat het om bijvoorbeeld bloed, urine, speeksel, ontlasting of uitstrijkjes. Zorgverleners vragen u soms om toestemming om uw gegevens en lichaamsmateriaal te mogen gebruiken voor iets anders dan uw behandeling. Deze vragenlijst gaat daarover.

Deze folder is bedoeld om u te informeren over het geven van toestemming. De vragen komen uit twee wetenschappelijke onderzoeken die in Duitsland zijn gedaan. Het ging er in die onderzoeken om welke informatie patiënten in Duitsland nodig hebben om een bewuste keuze te kunnen maken. Wij willen graag weten wat de Nederlandse patiënt nodig heeft om een keuze te maken om wel of geen toestemming te geven.

Door deze onderzoeken weten we dat goede informatie belangrijk is voor een patiënt. Daarom vragen wij u ook onderstaande informatie te lezen voordat u antwoord geeft op de vragen. De informatie gaat over wat er precies gevraagd wordt en wat voor u de gevolgen zijn als u toestemming geeft.

Het lezen van de informatie (folder) hoort dan ook bij het beantwoorden van de vragen. Bij een aantal onderdelen van de vragenlijst vragen we alleen antwoord te geven als u de informatie gelezen hebt.

Met dit onderzoek wil de Patiëntenfederatie weten hoe de leden van het panel denken over het gebruik van hun gegevens en lichaamsmateriaal. Een ander doel is om medewerkers in de zorg te informeren over informatie die patiënten belangrijk vinden.

Beste deelnemer,

In uw ziekenhuis worden zieken verzorgd en artsen opgeleid. Maar er vindt ook medisch onderzoek plaats. Zonder onderzoek kunnen behandelingen niet verbeterd worden. Zulk onderzoek is alleen mogelijk als de onderzoekers voldoende gegevens en lichaamsmateriaal hebben. Om te leren over een ziekte, moet lichaamsmateriaal van patiënten met deze ziekte worden onderzocht in het laboratorium. Het ziekenhuis vraagt u toestemming om uw lichaamsmateriaal en gegevens te mogen gebruiken.

**Wat houdt toestemming in?**
Voor uw behandeling in het ziekenhuis worden gegevens en soms ook lichaamsmateriaal gevraagd. Dat kan nu zijn of later in uw behandeling. We geven deze gegevens aan onderzoekers, die een onderzoek doen dat goed is gekeurd door een groep deskundigen. Dit kunnen onderzoekers zijn van uw eigen ziekenhuis of van buiten uw ziekenhuis.

Uw toestemming is vrijwillig en kan altijd door u worden ingetrokken. Als u toestemming geeft, vragen wij u bij ieder bezoek aan het ziekenhuis of u nog steeds toestemming geeft. Als u geen toestemming meer geeft, worden uw gegevens en lichaamsmateriaal verwijderd of vernietigd. Soms kan dat technisch niet meer. Of u wel of geen toestemming geeft, heeft voor u geen voordelen of nadelen. Toestemming geeft u altijd schriftelijk. Het ziekenhuis slaat uw keuze op en u kunt een kopie van uw toestemmingsformulier zelf thuis bewaren.

Lichaamsmateriaal wordt bewaard op het ziekenhuisterrein in biobanken. Hier zijn de omstandigheden geschikt voor het bewaren van dit materiaal. Het lichaamsmateriaal dat onderzoekers willen gebruiken, is niet meer nodig voor uw behandeling. Anders wordt dit materiaal (na enige tijd) vernietigd.

Onderzoekers krijgen de gegevens en het materiaal alleen als ze toestemming hebben voor hun onderzoek. Die toestemming wordt gegeven door een commissie van deskundigen die kijkt of het onderzoek goed wordt gedaan. De commissie is van het ziekenhuis. De commissie krijgt vooraf informatie over wat er gebeurt in het onderzoek. De commissie geeft de onderzoekers advies. Dit zorgt ervoor dat het lichaamsmateriaal en de gegevens nuttig worden gebruikt. En dat patiënten of burgers hier voordeel van hebben.

Voor een onderzoek worden uw medische gegevens bewerkt. Een onderzoeker kan dus niet zien wie u bent. Vanuit het ziekenhuis hebben maar een paar mensen toestemming om deze gegevens weer aan u als persoon te kunnen koppelen. In het onderzoek wordt niet uw naam of geboortedatum gebruikt, maar een ‘pseudoniem’. Zo’n pseudoniem bestaat uit een aantal cijfers en letters, bijvoorbeeld XP12475. Dit wordt ‘pseudonimisering’ genoemd. Daardoor is het bijna onmogelijk om gegevens uit het onderzoek aan u als persoon te koppelen. Bovendien is het niet toegestaan om pseudoniemen weer te vertalen naar de echte personen, als je daar geen toestemming voor hebt. De Autoriteit Persoonsgegevens kan iemand die dat toch doet een boete geven.

U wordt gevraagd om ‘brede’ toestemming te geven. Dat wordt gedaan omdat nog niet bekend is voor wat voor soort onderzoek uw gegevens gebruikt worden. Er is dus vooraf niet duidelijk wanneer, hoe en welke onderzoeken met uw gegevens gedaan worden. Dit heeft een reden: veel belangrijke onderzoeksvragen van de toekomst weten we nu nog niet. Met wat onderzoekers nu weten en kunnen, begrijpen zij hoe ziekten verlopen. Maar de techniek staat niet stil. Misschien kan met de technische mogelijkheden van de toekomst veel meer. Onderzoekers willen dan mogelijk uw gegevens en materiaal gebruiken met die technieken.

**Wat gebeurt er als u toestemming geeft?**1. Een commissie van deskundigen beoordeelt het onderzoek waarvoor uw gegevens gevraagd worden. Na goedkeuring worden de gegevens gekoppeld aan een pseudoniem. Daarna worden de gegevens met dat pseudoniem gegeven aan een onderzoeker. Onderzoekers en medewerkers van het ziekenhuis mogen gegevens niet doorgeven aan iemand die deze gegevens niet mag hebben. Zoals bijvoorbeeld werkgevers of verzekeraars. Dat is streng verboden.

2. Veel ziekten zijn voor een deel erfelijk. Het kan nodig zijn uw lichaamsmateriaal op die erfelijkheid te onderzoeken. We wijzen erop dat op dit moment ook met dit onderzoek naar erfelijkheid, niet zeker is of iemand ziek gaat worden. Met andere woorden: u kunt er dus niet van uit gaan dat, omdat u toestemming geeft, uw materiaal op erfelijke ziektes onderzocht wordt. En, wanneer dat wel gebeurt kan niet gezegd worden of u of uw (klein)kinderen ziek wordt.

3. veel belangrijke onderzoeken kunnen alleen worden gedaan als er veel gegevens beschikbaar zijn. Daarom worden gegevens en lichaamsmateriaal uit Nederland soms gedeeld met andere landen. Dat doen ook de onderzoekers uit uw ziekenhuis. Sommige van uw gegevens kunnen daarom aan een buitenlandse onderzoeker worden gegeven. Dit kan alleen als de commissie van deskundigen van het ziekenhuis daar toestemming voor geeft. Als dit gebeurt, worden uw gegevens helemaal anoniem gemaakt. Dat betekent dat de persoon van wie de gegevens zijn, helemaal niet bekend is. Ook wordt gekeken of de onderzoeker die de gegevens ontvangt, die gegevens veilig bewaart en gebruikt.

**Welke gegevens worden er gebruikt?**
De onderzoekers bepalen op basis van hun onderzoeksvraag welke gegevens zij nodig hebben. Het kan dus zijn dat u toestemming geeft om uw gegevens te gebruiken maar dat de onderzoekers niet alle gegevens ook echt gebruiken.

**Hoe worden uw gegevens en lichaamsmateriaal beveiligd?**1. iedereen in het ziekenhuis, die met uw gegevens of lichaamsmateriaal werkt, moet alle informatie geheim houden.

2. onderzoeksgegevens en lichaamsmaterialen worden op een andere plaats bewaard dan patiëntgegevens.

3. onderzoekers krijgen gegevens of lichaamsmateriaal alleen met een pseudoniem te zien. Er zijn maar een paar medewerkers van uw ziekenhuis die meer mogen. Zij hebben toestemming om gegevens of lichaamsmateriaal te koppelen aan een persoon. Zij kunnen dit ook technisch.

**Wat zijn de voordelen voor u?**Door uw toestemming helpt u mee aan het verbeteren van de wetenschap. Het kan leiden tot een nieuwe behandeling of bijvoorbeeld ook zorgen voor (meer) kennis over een ziekte. Dit heeft voordelen voor toekomstige patiënten. Wordt u nu behandeld in het ziekenhuis? Dan wordt daar ook gebruik gemaakt van resultaten van eerder onderzoek.

**Hoe wordt u geïnformeerd over resultaten?**Onderzoekers gebruiken de gegevens om iets te leren over gezondheid in het algemeen en niet over een individuele patiënt. Het kan zo zijn dat onderzoekers bij hun werk toch informatie vinden die in het bijzonder gaat over de gezondheid van 1 enkele persoon of zijn (klein)kinderen. Dit worden nevenbevindingen genoemd.

Normaal gezien zijn zulke resultaten niet precies genoeg om zeker te weten dat iemand ook echt ziek is of gaat worden. En dat is niet zeker genoeg om iemand ongerust te maken of een medische behandeling te starten. Er moeten dan andere soorten en soms preciezere onderzoeken gedaan worden. Daarom worden in Nederlandse ziekenhuizen patiënten meestal niet geïnformeerd over individuele resultaten.

Er zijn uitzonderingen. In Nederland* hebben deskundigen situaties beschreven waarin het informeren over resultaten wel moet gebeuren. Bijvoorbeeld als een onderzoeker denkt dat een persoon een ziekte heeft die behandeld of voorkomen kan worden. En wel op een manier die wetenschappelijk bewezen is. In die gevallen kan het dus zo zijn dat u geadviseerd wordt nader onderzoek te laten doen. Deze regels zijn ook op uw toestemming van toepassing
**Link naar de gedragscode ‘Verantwoord omgaan met lichaamsmateriaal ten behoeve van wetenschappelijk onderzoek’*

**Wat zijn de risico's voor u?**Een ziekenhuis slaat uw gegevens digitaal op. Dat gebeurt op een veilige manier. Alleen personeel dat toestemming heeft mag uw gegevens zien. Zij ondertekenen daarvoor een geheimhoudingsverklaring.

Een ziekenhuis kan nooit met 100% zekerheid zeggen dat niemand illegaal uw gegevens ziet. Vanwege de uitgebreide beveiligingsmaatregelen is de kans dat iemand uw gegevens inziet heel laag. En, het inzien van gegevens door iemand die geen toestemming heeft, is strafbaar voor de persoon die dat doet!

**Kunt u uw toestemming op een later tijdstip intrekken?**Uw toestemming kunt u altijd en zonder reden intrekken. Het intrekken van uw toestemming heeft geen nadelige gevolgen. Wanneer u uw toestemming intrekt, zullen uw onderzoeksgegevens vernietigd worden. Er zijn uitzonderingen. Het vernietigen van al eerder uitgegeven lichaamsmateriaal kan niet. Ook verwijderen van onderzoeksgegevens van onderzoeken die al bekend gemaakt zijn is niet mogelijk.
