# Supplementary material for: Secondary research use of personal medical data: attitudes from patient and population surveys in The Netherlands and Germany
Source: Eur J Hum Genet. 2020 Oct 1;29(3):495–502. doi: 10.1038/s41431-020-00735-3 (PMC7940390; doi:10.1038/s41431-020-00735-3)
Supplement: Supplementary file 2 — Survey „data donation“ for medical research [file 41431_2020_735_MOESM2_ESM.docx]

**Survey „data donation“ for medical research**

1. Assuming, in the future, your personal health information, such as your medical history, exam results, x-rays, etc. are stored online in a digital health record. In this case, would you agree that your personal health information to be shared anonymously and free of charge for medical research so that diseases can be better diagnosed and new treatments developed in the future?

**Scale:**

-well and truly

- rather

- rather not

- In no case

- I do not know / not specified

2. The case you have answered “rather not”/”in no case”

And why would you disagree that your personal health data is shared anonymously and free of charge for medical research? What reasons do you think against it?

**Open**

3. The case you have answered “well and truly”/”rather”:

And who would you share your personal health data for medical research with?

RANDOMIZING, MULTIPLEING POSSIBLE
- scientists at universities and public research institutes
- scientists from industry and private companies, e.g. the medical industry, biotech companies, etc.

- Other: PLEASE NOTE INDIVIDUALLY
- none of them
- do not know / no answer

4. The case you have answered “well and truly”/”rather”:

And how and for how long should your personal health data be used for medical research after your donation?

Only one answer allowed

- My personal health data should be used indefinitely for medical research.

- My personal health data is expected to be used for medical research for the next 5 years.

- I want to be asked for my consent to every single research project.

- do not know / no answer

5. For analytical purposes

Do you suffer from a chronic illness that causes you permanent medical treatment, such as diabetes, cancer or heart disease?

- Yes

- No

- do not know / no answer
