# Supplementary material for: Secondary research use of personal medical data: attitudes from patient and population surveys in The Netherlands and Germany
Source: Eur J Hum Genet. 2020 Oct 1;29(3):495–502. doi: 10.1038/s41431-020-00735-3 (PMC7940390; doi:10.1038/s41431-020-00735-3)
Supplement: Supplementary file 3 — Delen van uw data in het ziekenhuis [file 41431_2020_735_MOESM3_ESM.pdf]

(http://www.patientenfederatie.nl)
(http://www.patientenfederatie.nl)

Tekstgrootte: A A A

Via SSL-beveiligde verbinding

Delen van uw data in het ziekenhuis

In te vullen door Patiëntenfederatie Nederland - Marita Hassels

Vragenlijst Delen van uw data in het ziekenhuis

In het ziekenhuis wordt bij patiënten regelmatig bloed of urine afgenomen om te kijken of iemand iets mankeert. En worden er vragen gesteld over uw gezondheid. Misschien herkent u dit en is dit bij u ook al eens gedaan. Dit materiaal en medische gegevens kunnen vervolgens ook gebruikt worden voor iets anders dan uw behandeling, bijvoorbeeld voor wetenschappelijk onderzoek. Hiervoor moet in veel gevallen toestemming aan u worden gevraagd. Daarover gaat deze vragenlijst. We zijn heel benieuwd hoe u hierover denkt, ook als u hier geen ervaring mee heeft.

Als u al vaker met onze onderzoeken heeft meegedaan, dan ziet deze vragenlijst er iets anders uit dan u van ons gewend bent. Dat komt doordat dit onderzoek ook in Duitsland is uitgevoerd. De vragen moeten daarom zoveel mogelijk hetzelfde blijven, zodat goed kan worden vergeleken.

Alvast hartelijk dank voor uw deelname!

Bent u de afgelopen twee jaar in een ziekenhuis geweest voor behandeling of onderzoek? *

Verplichte vraag

- Ja
- Nee
- Weet ik niet

Is er de afgelopen twee jaar in een ziekenhuis lichaamsmateriaal bij u afgenomen, zoals bijvoorbeeld bloed, urine, speeksel, ontlasting, uitstrijkjes of een biopt? *

Verplichte vraag

- Ja
- Nee
- Weet niet

Heeft u wel eens meegedaan aan een wetenschappelijk onderzoek naar een ziekte, of behandeling, waarvoor u specifieke vragenlijsten heeft ingevuld of lichaamsmaterialen heeft afgestaan?

- Ja
- Nee
- Weet ik niet

Artsen en onderzoekers die gegevens en lichaamsmateriaal van patiënten verzamelen tijdens de behandeling, kunnen deze ook gebruiken om nieuw onderzoek mee te doen.

Is u bij een bezoek aan een ziekenhuis weleens gevraagd om toestemming te geven voor het gebruiken van uw medische gegevens en/of lichaamsmateriaal voor wetenschappelijk onderzoek? *

Verplichte vraag

- Ja
- Nee
- Weet ik niet

Wanneer werd u dit gevraagd?

- Minder dan een jaar geleden
- Langer dan een jaar geleden, maar korter dan 2 jaar
- Langer dan 2 jaar geleden

Heeft u toen toestemming gegeven om uw gegevens of lichaamsmateriaal te gebruiken? *

Verplichte vraag

- Ja
- Nee

Stel dat u in het ziekenhuis wordt gevraagd om toestemming te geven voor het gebruiken van uw bestaande medische gegevens en/of lichaamsmateriaal voor wetenschappelijk onderzoek. Zou u toestemming geven? *

Verplichte vraag

- Ja
- Nee
- Weet ik niet

Waarom heeft u toestemming gegeven voor het gebruik van uw gegevens of materiaal?

Meerdere antwoorden mogelijk

- Ik help graag andere patiënten met dezelfde aandoening.
- Ik ben mijn artsen dankbaar en geef mijn toestemming juist om hen te helpen.
- Ik help graag andere patiënten, het maakt mij niet uit welke ziekte ze hebben.
- Ik ben bang dat ik nadelen ondervind of dat mijn behandeling eronder lijdt als ik mijn toestemming niet geef.
- Ik hoop dat ik in de toekomst zelf persoonlijk voordeel heb van het onderzoek met mijn gegevens en lichaamsmateriaal.
- Ik heb er niet over nagedacht.

- [ ] Ik ken andere patiënten die ermee instemden en dit overtuigde me om ook toestemming te geven.
- [ ] Ik weet dat ik voordeel gehad heb van medisch onderzoek, daarom vind ik dat ik in ruil voor dat voordeel ook mee moet helpen voor toekomstige patiënten.
- [ ] Ik ondersteun graag medisch onderzoek in het algemeen.
- [ ] Omdat ik zelf patiënt ben, voel ik me verbonden met toekomstige patiënten en zou ik graag iets voor hen willen doen.

Waarom heeft u geen toestemming gegeven voor het gebruik van uw gegevens of materiaal?

≡ Meerdere antwoorden mogelijk

- [ ] Ik verwacht zelf geen voordeel van onderzoek met mijn gegevens en lichaamsmaterialen.
- [ ] Ik ben bang dat het niet goed voor mij of mijn behandeling is als ik toestemming geef.
- [ ] Ik wil niet dat mijn gegevens en lichaamsmaterialen worden gebruikt voor onderzoek naar andere ziekten.
- [ ] Ik ben bang dat mijn gegevens niet voldoende worden beschermd.
- [ ] Ik denk niet dat ik tot nu toe voordeel heb gehad van medisch onderzoek .
- [ ] Ik weet niet genoeg over wat er wordt gedaan met mijn gegevens en lichaamsmaterialen.
- [ ] Ik heb er niet over nagedacht.
- [ ] Ik weet niet precies wie het onderzoek uitvoert.

Waarom zou u toestemming geven voor het gebruik van uw gegevens of materiaal?

≡ Meerdere antwoorden mogelijk

- [ ] Ik zou bang zijn dat ik nadelen ondervind of dat mijn behandeling eronder zou lijden als ik mijn toestemming niet geef.
- [ ] Ik ben mijn artsen dankbaar en zou mijn toestemming geven juist om hen te helpen.
- [ ] Ik help graag andere patiënten met dezelfde aandoening.
- [ ] Ik ken andere patiënten die ermee instemden en dit zou mij overtuigen om ook toestemming te geven.
- [ ] Ik weet dat ik voordeel gehad heb van medisch onderzoek, daarom vind ik dat ik in ruil voor dat voordeel ook mee moet helpen voor toekomstige patiënten.
- [ ] Ik ondersteun graag medisch onderzoek in het algemeen.
- [ ] Ik help graag andere patiënten, het maakt mij niet uit welke ziekte ze hebben.
- [ ] Ik heb er nog niet over nagedacht.
- [ ] Omdat ik in de toekomst zelf persoonlijk voordeel kan hebben van het onderzoek met mijn gegevens en lichaamsmateriaal.
- [ ] Omdat ik zelf patiënt ben, voel ik me verbonden met toekomstige patiënten en zou ik graag iets voor hen willen doen.

Waarom zou u geen toestemming geven voor het gebruik van uw gegevens of materiaal?

≡ Meerdere antwoorden mogelijk

- [ ] Ik wil niet dat mijn gegevens en lichaamsmaterialen worden gebruikt voor onderzoek naar andere ziekten.
- [ ] Ik verwacht zelf geen voordeel van onderzoek met mijn gegevens en lichaamsmaterialen.
- [ ] Ik weet niet genoeg over wat er wordt gedaan met mijn gegevens en lichaamsmaterialen.
- [ ] Ik denk niet dat ik tot nu toe voordeel heb gehad van medisch onderzoek.

- [ ] Ik heb er nog niet over nagedacht.
- [ ] Ik ben bang dat mijn gegevens niet voldoende worden beschermd.
- [ ] Ik ben bang dat het niet goed voor mij of mijn behandeling is als ik toestemming geef.
- [ ] Ik weet niet precies wie het onderzoek uitvoert.

Weet u wat er op dit moment gebeurt met uw overgebleven lichaamsmateriaal/gegevens uit uw huidige onderzoeken bij ziekenhuizen? * Verplichte vraag

- ( ) Ja
- ( ) Nee

U kunt hier aangeven wat er volgens u gebeurt met uw overgebleven lichaamsmateriaal/gegevens uit uw huidige onderzoeken bij ziekenhuizen.

Tot nu toe ging het over het vragen van toestemming voor het gebruik van uw gegevens voor wetenschappelijk onderzoek.

Gegevens kunnen ook worden gebruikt voor andere soorten onderzoek, bijvoorbeeld voor het maken van medicijnen, hulpmiddelen of apps door commerciële organisaties. Zou u toestemming geven voor het gebruiken van uw gegevens voor die doelen? * Verplichte vraag

- ( ) Ja
- ( ) Nee
- ( ) Dat hangt af van de organisatie en/of het doel van deze organisatie

Kunt u uw antwoord toelichten?

Soms wordt een behandeling niet vergoed door de zorgverzekeraar, maar kunt u de behandeling wel vergoed krijgen als u uw gegevens en/of lichaamsmateriaal beschikbaar stelt voor onderzoek.

Heeft u uw gegevens en/of lichaamsmateriaal wel eens beschikbaar gesteld, omdat u dan de behandeling vergoed kreeg? *

❗ Verplichte vraag

- Ja
- Nee
- Weet ik niet

U kunt hier uw antwoord toelichten.

Voor veel ontwikkelingen en onderzoek is het belangrijk dat mensen hun medische gegevens en lichaamsmateriaal beschikbaar stellen. Als u moet beslissen of u hier wel of niet aan meewerkt, dan wilt u hier natuurlijk wel een goede uitleg over.

Daarvoor is op dit moment een folder in ontwikkeling. Wilt u de folder lezen en hierover een aantal vragen beantwoorden? Het lezen van de folder en het beantwoorden van de vragen kost ongeveer 10 minuten.

Bent u panellid en heeft u nu geen tijd om de folder door te lezen, dan kunt u op een willekeurig ander moment de vragenlijst weer openen en dan op dezelfde plek verdergaan.

- Ja
- Nee

Via onderstaande link kunt u de folder openen.

Klik hier om de folder te lezen (https://www.patientenfederatie.nl/Documenten/Folder-20190910.pdf)

Als u de folder gelezen heeft, kunt u doorgaan met het invullen van de vervolgvragen.
Heeft u problemen met het openen van de folder, dan kunt u terug naar de vorige vraag om daar 'nee' in te vullen. U slaat dan het onderdeel met vragen over de folder over.

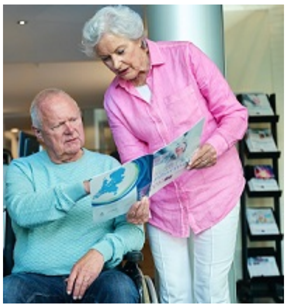

Wat vindt u van de lengte van de folder?

- Te kort
- Te lang

- ○ Precies goed

Begrijpt u de informatie die in de folder staat? * Verplichte vraag

- ○ Ja
- ○ Niet alles
- ○ Nee

U geeft aan de folder niet of niet helemaal te begrijpen. Wat is de reden dat u niet alles begrijpt? Meerdere antwoorden mogelijk

- ☐ Ik begrijp de technische informatie niet
- ☐ Te veel informatie
- ☐ Te weinig uitleg
- ☐ Te lang
- ☐ Taal is te ingewikkeld
- ☐ Ik heb te snel gelezen
- ☐ Ik ben niet geïnteresseerd in de inhoud

Hoe kunnen we de informatie in de folder verbeteren? Wat mist er, wat kan beter?

We willen graag weten welke informatie in de folder voor u het meest en het minst duidelijk is.

Hieronder worden enkele uitspraken gedaan. Kunt u bij iedere uitspraak aangeven of deze klopt met de informatie uit de folder?

	Dit staat zo in de folder	Dit staat niet zo in de folder	Ik weet het niet zeker
Mijn gegevens en lichaamsmateriaal worden alleen gebruikt bij onderzoeken die te maken hebben met mijn ziekte of klacht.	○	○	○
Ik kan mijn toestemming om de gegevens op te slaan op elk moment intrekken. Gegevens en lichaamsmateriaal zullen worden verwijderd voor zover dit technisch mogelijk is.	○	○	○
Mijn gegevens en lichaamsmateriaal kunnen door mijn ziekenhuis worden doorgegeven aan onderzoekers buiten het ziekenhuis. Maar alleen bij belangrijk of nuttig medisch onderzoek dat is goedgekeurd door een onafhankelijke commissie van deskundigen.	○	○	○

	Dit staat zo in de folder	Dit staat niet zo in de folder	Ik weet het niet zeker
Als ik toestemming geef dat mijn gegevens mogen worden gebruikt, kan ik deze toestemming niet intrekken.	○	○	○

Hieronder worden wederom enkele uitspraken gedaan. Kunt u bij iedere uitspraak aangeven of deze klopt met de informatie uit de folder?

	Dit staat zo in de folder	Dit staat niet zo in de folder	Ik weet het niet zeker
Mijn gegevens en lichaamsmateriaal worden alleen gebruikt in het ziekenhuis dat mijn toestemming heeft gevraagd.	○	○	○
Mijn gegevens en lichaamsmateriaal kunnen worden gebruikt voor ieder ander medisch onderzoek.	○	○	○
Mijn bestaande gegevens worden voor het onderzoek anoniem gemaakt of van een pseudoniem voorzien. De onderzoekers weten daarom niet dat het om mij gaat.	○	○	○
Als tijdens onderzoek met mijn medische gegevens en lichaamsmateriaal iets is gevonden, krijg ik dat niet te weten. Behalve als het gaat om een ernstige ziekte die bij mij geconstateerd wordt waarvan bewezen is dat die behandeld of voorkomen kan worden.	○	○	○

Welke uitspraak vindt u juist? Met het onderzoek met mijn medische gegevens en lichaamsmateriaal:

≡ Meerdere antwoorden mogelijk

- ☐ Zou ik een direct persoonlijk voordeel voor mijn gezondheid kunnen hebben.
- ☐ Kunnen toekomstige patiënten mogelijk voordelen hebben voor hun gezondheid.
- ☐ Zullen alleen onderzoek en de medische wetenschap voordeel hebben.

In Nederland worden patiënten niet geïnformeerd over (individuele) resultaten die uit het onderzoek met uw gegevens en lichaamsmateriaal komen. Ook wordt niet aangegeven of uw gegevens wel of niet zijn gebruikt voor onderzoek.

Dit is alleen anders als een onderzoeker, die uw materiaal gebruikt heeft, iets ernstigs vindt bij u dat behandeld of voorkomen kan worden. Vindt u dit juist?

- ○ Ja
- ○ Nee
- ○ Ik weet het niet zeker

Kunt u uw antwoord toelichten?

Eerder hebben we u gevraagd of u toestemming geeft of zou geven voor het gebruiken van uw medische gegevens en lichaamsmateriaal voor wetenschappelijk onderzoek. Denkt u hier anders over nu u de folder gelezen heeft? *

Verplichte vraag

- Ja
- Nee, ik denk er nog hetzelfde over
- Weet ik niet

U denkt er nu anders over. Welke uitspraak is op u van toepassing?

- Ik gaf eerder geen toestemming, maar nu wel
- Ik gaf eerder wel toestemming, maar nu niet meer

Wat heeft ervoor gezorgd dat u nu wel toestemming geeft?

Meerdere antwoorden mogelijk

- Ik kan mijn toestemming altijd weer intrekken
- Er is een commissie van deskundigen die kijkt of onderzoek goed wordt gedaan
- Mijn lichaamsmateriaal kan nu nog nuttig zijn, terwijl er anders niets meer mee gedaan wordt
- In het onderzoek wordt mijn naam of geboortedatum niet gebruikt, maar een pseudoniem
- Ik kan in de toekomst zelf persoonlijk voordeel hebben van het onderzoek met mijn gegevens en lichaamsmateriaal.
- Ik kan medisch onderzoek in het algemeen ondersteunen.
- Ik kan zo andere patiënten helpen.
- Ik heb zelf voordeel van medisch onderzoek, daarom moet ik in ruil voor dat voordeel ook mee helpen voor toekomstige patiënten.
- Omdat ik hier geen nadeel van ondervind
- Mijn gegevens worden voldoende beschermd
- Weet ik niet

Wat heeft ervoor gezorgd dat u nu geen toestemming meer geeft?

Meerdere antwoorden mogelijk

- Omdat ik niet krijg te horen voor wat voor soort onderzoek mijn gegevens worden gebruikt.
- Mijn gegevens kunnen aan onderzoekers in het buitenland worden gegeven
- Mijn gegevens worden digitaal opgeslagen
- Ik zie geen voordeel van onderzoek met mijn gegevens en lichaamsmaterialen

- ☐ Ik weet toch niet genoeg over wat er wordt gedaan met mijn gegevens en lichaamsmaterialen
- ☐ Ik weet niet precies wie het onderzoek uitvoert
- ☐ Ik wil niet dat mijn gegevens en lichaamsmaterialen worden gebruikt voor onderzoek naar andere ziekten
- ☐ Ik ben bang dat mijn gegevens niet voldoende worden beschermd.
- ☐ Ik ben bang dat het niet goed voor mij of mijn behandeling is als ik toestemming geef.
- ☐ Weet ik niet

Waarom denkt u hier nog steeds hetzelfde over?

≡ Meerdere antwoorden mogelijk

- ☐ De folder bevestigt wat ik al dacht
- ☐ Omdat ik de folder niet snap
- ☐ Omdat ik dit allemaal al wist
- ☐ Weet ik niet

U kunt hier uw antwoord nog verder toelichten.

Heeft u nog andere opmerkingen over het delen van gegevens en lichaamsmateriaal voor wetenschappelijk onderzoek? Dan kunt u die hieronder kwijt.

Achtergrondvragen

Tot slot willen we u enkele achtergrondvragen voorleggen.

Hoe vaak bent u in het afgelopen jaar bij een zorgverlener geweest voor behandeling, consult of opname?

- ○ Niet
- ○ 1 keer
- ○ 2 tot 5 keer
- ○ 6 tot 10 keer
- ○ Vaker dan 10 keer

Hoe belangrijk zijn de volgende dingen voor u? *

Verplichte vraag

	Helemaal niet belangrijk	Niet belangrijk	Redelijk onbelangrijk	Redelijk belangrijk	Belangrijk	Erg belangrijk
Macht: accepteren dat iemand anders een beslissing neemt, rijkdom, hoe ik word gezien. *	○	○	○	○	○	○
Succes: succes hebben, ergens goed in zijn, de beste willen zijn, het belangrijkste willen zijn. *	○	○	○	○	○	○
Eigen regie: controle, creativiteit, vrijheid, onafhankelijk, nieuwsgierig, het kiezen van uw eigen doelen *	○	○	○	○	○	○
Genot: plezier, genieten van het leven *	○	○	○	○	○	○
Uitdaging: gedurfd, een afwisselend leven, een spannend leven *	○	○	○	○	○	○

Hoe belangrijk zijn de volgende dingen voor u? *

Verplichte vraag

	Helemaal niet belangrijk	Niet belangrijk	Redelijk onbelangrijk	Redelijk belangrijk	Belangrijk	Erg belangrijk
Veiligheid: gezinsveiligheid, nationale veiligheid, sociale orde, niemand iets verschuldigd zijn. *	○	○	○	○	○	○

	Helemaal niet belangrijk	Niet belangrijk	Redelijk onbelangrijk	Redelijk belangrijk	Belangrijk	Erg belangrijk
Tevredenheid: accepteer mijn positie in het leven, bescheiden, respect voor traditie, gematigd *	○	○	○	○	○	○
Sociaal/menselijk: behulpzaam, eerlijk, vergevingsgezind, trouw, verantwoordelijk *	○	○	○	○	○	○
Houden aan algemene gedragsregels: beleefdheid, gehoorzaamheid, zelfdiscipline, respect voor ouders en oudere mensen, etiquette *	○	○	○	○	○	○
Samenleven op de wereld: tolerant, sociale rechtvaardigheid, gelijkheid, een wereld in vrede, een wereld vol schoonheid, eenheid met de natuur, bescherming van het milieu *	○	○	○	○	○	○

Bent u een man of een vrouw? *

Verplichte vraag

- ○ Man
- ○ Vrouw
- ○ Anders

Wat is uw geboortedatum?

Antwoord: Dag ▾ - Maand ▾ - Jaar ▾

Heeft u één of meer chronische aandoeningen?

Meerdere antwoorden mogelijk

- ☐ Geen
- ☐ Astma/COPD

- [ ] Reumatische aandoening
- [ ] Darmaandoening
- [ ] Diabetes
- [ ] Hart- en Vaatziekten
- [ ] Kanker
- [ ] Lichamelijke beperking
- [ ] Maagklachten
- [ ] Psychische problemen (GGZ)
- [ ] Verstandelijke beperking
- [ ] Anders, namelijk:

In welke provincie woont u?

Antwoord: Maak een keuze...

Wat is de hoogste opleiding die u heeft afgerond?

Antwoord: Maak een keuze...

Wie is uw zorgverzekeraar?

Antwoord: Maak een keuze...

Bent u bereid om eventueel mee te werken aan een interview over het onderwerp van deze vragenlijst?

- Ja
- Nee

Bent u bereid vaker mee te werken aan vragenlijsten van de Patiëntenfederatie Nederland?

Als u vaker mee wilt werken aan vragenlijsten, kunt u deelnemen aan het Zorgpanel van de Patiëntenfederatie Nederland. U ontvangt dan een aantal keer per jaar per e-mail een uitnodiging om aan een vragenlijst mee te werken.

- Ja
- Doe ik al aan mee
- Nee

Persoonsgegevens

Uw persoonsgegevens worden vertrouwelijk behandeld. Graag uw e-mailadres vermelden wanneer we nogmaals mogen benaderen. Graag ook uw naam en telefoonnummer vermelden wanneer u beschikbaar bent voor een interview.

Naam	
E-mail	
Telefoonnummer	
Woonplaats	

U bekijkt deze vragenlijst in de voorbeeldweergave. U kunt daarom geen resultaten verzenden!

Via SSL-beveiligde verbinding
